# Assessment of Clinical Analgesic Levels and Serum Biomarkers in Patients with Rheumatoid Arthritis: A Randomized Controlled Trial Comparing the Efficacy of Diclofenac and Methotrexate Combined Therapy with Extracorporeal Shockwave Therapy

**DOI:** 10.1155/2024/6687987

**Published:** 2024-08-21

**Authors:** Mei Zhang, Zhongyuan Ma, Rinkiko Suguro, Menglin Zhu, Esther Xinyi Chen, Xin Dong, Meixiu Chen, Linling Cheng, Bolun Su, Yizhun Zhu

**Affiliations:** ^1^ School of Pharmacy and State Key Laboratory for the Quality Research of Chinese Medicine Macau University of Science and Technology, Av. Wai Long, Macau 999078, China; ^2^ Department of Cardiothoracic Surgery Zhuhai People's Hospital Zhuhai Hospital Affiliation with Jinan University, Zhuhai, Guangdong 519000, China; ^3^ Faculty of Medicine Macau University of Science and Technology, Av. Wai Long, Macau 999078, China; ^4^ Macau Institute of Systems Engineering Macau University of Science and Technology, Taipa, Macau 999078, China; ^5^ Semiahmoo Secondary School, 1785 148 St, Surrey, BC, Canada; ^6^ Faculty of Chinese Medicine Macau University of Science and Technology, Macau, China

## Abstract

**Background:**

Rheumatoid arthritis (RA) is one of the most common forms of arthritis. Extracorporeal shockwave therapy (ESWT) has been identified as a viable alternative therapeutic approach in light of the present protracted clinical course of pharmacological treatment, and changes in levels of marker proteins in the blood samples of RA patients can be utilized to assess treatment outcomes.

**Methods:**

A randomized controlled trial was conducted involving forty patients diagnosed with rheumatoid arthritis (RA) who were assigned randomly to two groups. The first group received a combination of diclofenac and methotrexate (MTX) consisting of 25 mg of diclofenac administered thrice daily and 15 mg of MTX administered once weekly. Individual follow-up assessments were carried out after 7 and 14 days. Meanwhile, patients in the second group underwent two sessions of Extracorporeal Shockwave Therapy (ESWT), with a 7-day interval between sessions. Evaluations were conducted on day 7 and day 14. Patients who displayed pain control and stability were advised to continue the treatment, whereas those who had inflammation and discomfort were administered specific medications, and their progress was closely monitored until day 28. Blood samples were collected from both groups prior to treatment, after the first treatment, and after the second treatment. Four marker proteins (NRP-1, CELF-6, COX-2, and RGS-1) and two inflammatory cytokines (IL-6 and IL-17) were measured using western blot and RT-PCR techniques. A statistical analysis was conducted on the levels of specific proteins and inflammatory factors before and after treatment to evaluate its impact.

**Result:**

Both groups exhibited statistically significant differences in the serum level of target biomarkers before and after the intervention. However, the ESWT group demonstrated a more noticeable effect, while the diclofenac + MTX group exhibited a delayed anti-inflammatory effect compared to ESWT.

**Conclusion:**

Both treatments significantly improved joint function, relieved pain, and reduced inflammation in patients. However, ESWT demonstrated a more prominent clinical analgesic effect compared to the combination treatment of diclofenac and MTX. Furthermore, ESWT produced a more immediate and noteworthy anti-inflammatory impact by regulating NRP-1 expression, a trophic factor receptor that facilitates vascular endothelial cell migration and tissue repair through angiogenesis, and regulating RGS-1 to limit inflammatory signal transmission and immune cell activation.

## 1. Introduction

Rheumatoid arthritis (RA) is a systemic autoimmune disease [1] manifested primarily as pain and swelling in multiple joints and other organs of the body [2, 3], which profoundly impacts mobility, quality of life, and life expectancy [4]. In the past decades, the prevalence of RA has surged alongside population aging [5], making early intervention of RA and delay of its progression crucial.

Innovative treatment strategies have the potential to improve early diagnosis and medical intervention; however, the current state of RA care is inadequate due to expensive medications that hinder accessibility to medical care [6]. The prevailing therapeutic approach centers around antirheumatic drugs (DMARDs), with a usual treatment cycle of 3–6 months. This long-term medication imposes not only a financial strain, particularly for patients with poorly managed and treatment-resistant RA, but also increases the burden on liver and kidney function in vulnerable patients, thus worsening their metabolic decline.

Recent developments in the medical field aim to accelerate the acquisition of effective medical treatments for patients. Extracorporeal shockwave therapy (ESWT) is a noninvasive technique that utilizes high-energy shockwaves at the site of pain to encourage healing and tissue regeneration. Consequently, it mitigates chronic inflammation and relieves pain [7]. Building on the success of ESWT in treating soft tissue injuries such as ankle arthritis [7] and knee tendinopathy [8], recent advancements are exploring the particular molecular mechanisms of tissue regeneration and healing stimulated by shockwaves. There is a focus on understanding the processes that enable shockwaves to promote tissue restoration to improve efficacy in the treatment of musculoskeletal disorders.

In a previous study, we confirmed that NAV2 levels were significantly increased in synovial fibroblasts from RA patients and in synovial tissue from rats with adjuvant-induced arthritis (AIA) [9]. Additional studies are revealing other markers of RA, such as sNRP-1 [10], CELF-6 [10], COX-2 [11], and rgs-1 [12], with subsequent animal model studies suggesting that inhibiting the expression of these biomarker proteins could effectively alleviate inflammation and delay disease progression in RA [13].

**Figure 1:**
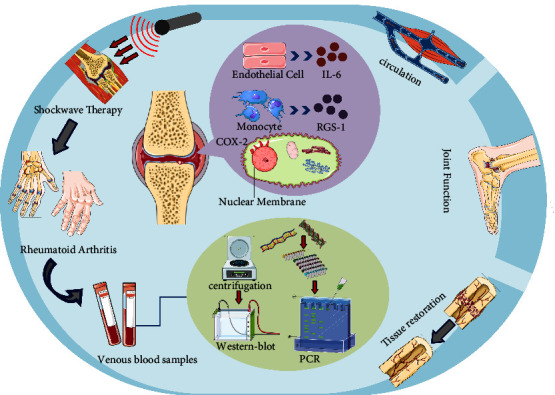
Efficacy of shockwave therapy in RA patients and the mechanism.

Based on the existing experimental evidence, this study analyzes changes in protein levels and inflammatory factors in the blood of RA patients before and after shockwave therapy. The study aims to confirm the effectiveness of ESWT and to propose new therapeutic targets for RA treatment. The hypothesis is that ESWT can alleviate RA symptoms by decreasing the inflammatory response and impacting specific protein and cytokine levels (Figure 1). The null hypothesis (H0) states that there is no statistically significant difference in the concentration and expression of serum protein markers between patients who received EWST treatment and those who were treated with diclofenac and MTX.

## 2. Materials and Methods

### 2.1. Subjects

During the study, eligible patients were invited to participate in collaboration with the Macau University of Science and Technology Hospital. Of the 47 participants enrolled during the patient recruitment phase, some reported limited motion in their wrists and knees accompanied by pain. Five individuals were excluded for not meeting the inclusion criteria, while two others were excluded for not signing the informed consent form. During the recruitment process, we ensured transparent and impartial communication with all participants regarding the study's objectives and requirements. Dr. Zhang Mei oversaw patient recruitment for the clinical research study, which took place from April 20 to October 12, 2021. The trial began on December 6, 2021, and consisted of two interventions. Subsequently, two assessments were conducted on December 12 and December 18, respectively.

#### 2.1.1. Exclusion and Inclusion Criteria

The target population comprised RA patients exhibiting at least 1 joint with definite clinical synovitis (swelling) not attributable to any other condition.

We included patients with a confirmed diagnosis of RA and agreed to receive ESWT; RA diagnosis adhered to the diagnostic criteria of the American College of Rheumatology (ACR), which is structured as a score-based algorithm (sum score of categories A–D ≥6/10 is needed for definitive classification as RA) [14] (Table 1).

Exclusion criteria include other types of arthritis or one or more of the following conditions:

- Infection at the treatment site, hematoma
- BP <90/60 mmHg, HR <50 beats/min
- Coagulation dysfunction
- Severe cardiopulmonary, hepatic, and renal insufficiency
- Psychiatric disorder, unable to clearly express pain, NRS score
- Participation in other clinical studies within three months
- Any reason deemed by the investigator to be inappropriate for participation in this trial

Prior to their participation, all enrolled patients willingly provided signed informed consent, and the study was approved by the ethics committee of Macau University Hospital.

#### 2.1.2. Randomization of Groups

To ensure strict randomization, we used a rigorous methodology. We created a block randomization list with a computerized random-number generator. After the initial baseline assessment, an unbiased researcher, with no involvement in the intervention or data evaluation, assigned patients to either the Extracorporeal Shock Wave Therapy (ESWT) group or the Diclofenac + Methotrexate (MTX) group, based on the systematic alternation of odd and even numbers. This led to an even allocation of individuals, with an equitable 50/50 split between the shockwave and Diclofenac + MTX subgroups. Furthermore, stratified randomization was employed, considering critical baseline variables such as disease severity and previous medication use, ensuring that these covariates were balanced and thus reducing the potential for confounding effects.

### 2.2. Shockwave Intervention

Shockwave treatment involved the use of a Piezo Wave instrument (Richard-Wolf; German). ESWT interventions were administered by a technician, and the specific parameters are listed in Table 2. The procedure was conducted in the physiotherapy facility at the Macau University of Science and Technology Hospital.

In the ESWT group, patients underwent two treatments each week according to a structured protocol. They were initially placed in a reclined position, either sitting or lying down, and then manual techniques were employed to determine the treatment region and depth, usually assessed through participant feedback. Ultrasound gel was applied to the skin to serve as a coupling agent, reducing energy loss at the interface. The shockwave probe was carefully positioned on the trigger point or slowly maneuvered around the joint, steering clear of nerves and vessels. Each treatment session lasted 30 minutes.

During which the treatment probe administers about 2000 pulses. In the beginning, to accurately locate the treatment region, the device was set to low intensity (0.15–0.25 mJ/mm^2^, depending on individual tolerance) and low frequency (less than 4 Hz). Biomechanical feedback was employed when necessary to optimize outcomes. After identifying the optimal treatment position, we gradually increased the frequency to 8 Hz for muscle treatment and 4 Hz for tendon treatment. The energy intensity for muscle therapy was raised to the highest tolerable level, while only half of the patient's maximum tolerance was used for the tendons.

It should be noted that this overview presents a general framework of the procedures employed in shockwave therapy. Treatment protocols are tailored according to the specific condition being treated.

### 2.3. Medication Intervention

Before the trial began, all participants received detailed instructions on the medication administration process. They were advised to strictly adhere to the given dosage and frequency of the study. MTX: 2.5 mg/tablet, 6 tablets of 15 mg, once a week. Diclofenac: 25 mg/tablet, 1 tablet of 25 mg, 3 times a day. Trained clinical staff dispensed the investigational drug provided by Sandoz Inc., which is a global generic pharmaceuticals company and a subsidiary of Novartis, to participants during each scheduled visit. The medication supplier, Sandoz Inc., ensured that all medications were packaged and labeled in accordance with regulatory standards, maintaining consistent quality throughout the trial period.

### 2.4. Documentation, Evaluation, and Management of Adverse Reactions

#### 2.4.1. Documentation and Evaluation of Adverse Reactions

If an adverse event occurs during the study, the investigator has the duty to immediately evaluate the nature of the event and administer the required therapeutic measures to safeguard the subject's rights.

At each clinical visit, the investigator must question the subjects about any adverse events and document both the directly observed and self-reported adverse events in an electronic case report form. All adverse events, including serious adverse events, require ongoing monitoring by the investigator and multiple follow-up visits until the event is resolved. Furthermore, documenting the severity and any changes in the patient's adverse reactions during each follow-up session serves to evaluate whether the patient is suitable for continued participation in the clinical trial. It is essential to adhere to these guidelines when conducting clinical research to ensure patient safety and the reliability of results.  Table 3 classifies potential adverse reactions into three levels of severity.

#### 2.4.2. Correlation of Adverse Reactions with Medical Operation Treatment

The researchers would assess the relevance of each adverse event to the medical operation. The assessment of causality would follow the definitions in the above table. In the reporting process, including the management of records, probable correlation, likely correlation, and definite correlation are all judged to be relevant.

### 2.5. Clinical Indicator Measurement

During this randomized controlled trial, the main objective was to evaluate inflammation markers and their expression and plasma concentrations. To conduct this individual-level assessment, quantitative real-time fluorescence polymerase chain reaction (RT-qPCR) and western blot tests were conducted at baseline and periodically after each treatment initiation. The secondary outcomes were evaluated utilizing the Patient Health Questionnaire (PHQ) and the Generalized Anxiety Disorder (GAD-7) for depression and anxiety scoring, respectively, and a numerical rating scale (NRS) was used to quantify the progress made in reducing pain intensity. Blinded assessors, unaware of the treatment allocation, conducted the evaluations simultaneously with the primary outcome measure.

#### 2.5.1. Pain Level Assessment

Patients' joint pain levels were assessed pre- and posttreatment through a numerical rating scale (NRS), where pain severity was graded on a scale of 0–10 instead of descriptive words. A straight line was divided into ten uniform segments, and patients were asked to circle the number that best represented the severity of pain they had felt within the previous 24 hours. Absence of pain is indicated by 0, whereas mild pain that does not affect sleep is indicated by 1–3. A score of 4–6 represents moderate pain, while 7–9 signifies severe pain that disrupts sleep. A score of 10 corresponds to extremely severe pain.

#### 2.5.2. Anxiety and Depression Scoring

In terms of anxiety and depression levels, individuals diagnosed with RA have a higher prevalence of depression, ranging from 14–48%, as a result of the disease's anticipated and unforeseen symptoms, treatment side effects, and other factors, when compared to the general population. Notably, research suggests that anxiety and depression impact approximately 30% of patients with rheumatoid arthritis (RA), with a higher incidence among females.

This immune-mediated disease demonstrates an upregulation of proinflammatory cytokines, including IL-6, as well as other blood biomarker proteins, which may indirectly intensify exhaustion and discomfort, leading to anxiety and depressive symptoms. Therefore, monitoring the psychological well-being of clinical trial participants is critically important.

#### 2.5.3. Efficacy Criteria

We divided treatment efficacy into 3 tiers as follows:

  Cure standard: pain disappears and is maintained
  Effective criterion: after treatment, the patient's pain is relieved by more than 50%, and the ability to move is significantly better than before treatment
  Ineffective criterion: no chronic pain relief after treatment

### 2.6. Experimental Methodology

#### 2.6.1. Quantitative Real-Time Fluorescence PCR Analysis

*(1) Total mRNA Extraction*. Total RNA was isolated from human whole blood samples using the Total RNA extraction kit R1201 (ZYMO RESEARCH) following the manufacturer's instructions. Briefly, a 1 : 1 ratio of DNA/RNA Shield (2X concentrate) to whole blood was mixed thoroughly. Proteinase K was added, and the mixture was incubated at room temperature. After Proteinase K treatment, isopropanol was added, and the sample was centrifuged through a Zymo-Spin™ IIICG Column. The column was washed with RNA Recovery Buffer, and the flow-through was collected for ethanol precipitation. The RNA was further purified using a Zymo-Spin™ IC Column and DNase I treated to remove genomic DNA contamination. The RNA was eluted in DNase/RNase-Free Water and quantified for downstream applications.

*(2) Reverse Transcription*. For reverse transcription, 600 ng of total RNA was used with 5 × RT Master Mix in a final volume of 15 *μ*l. The reaction conditions were set to 35°C for 15 minutes, 85°C for 5 seconds, followed by a hold at 4°C for 1 hour. The cDNA generated was diluted threefold with DEPC water prior to quantitative PCR analysis.

*(3) PCR*. The quantitative PCR was performed using a 2 × SYBR Green PCR master mix. Each 20 *μ*l reaction contained 10 *μ*l of 2 × SYBR Green, 0.4 *μ*l of each forward and reverse primer, 7.2 *μ*l of DEPC water, and 2 *μ*l of cDNA. The thermocycling conditions were initiated with a predenaturation step at 95°C for 10 minutes, followed by 40 cycles of 95°C for 15 seconds and 60°C for 45 seconds. A melting curve analysis was conducted starting at 55°C for 15 seconds, 65°C for 5 seconds, with a gradual increase to 95°C to ensure the specificity of the amplified product.

Detailed sequences of the primers are presented in Table 4. This methodology allowed for the quantitative comparison of mRNA expression levels of clinical analgesic biomarkers in patients receiving either combined therapy of diclofenac and methotrexate or extracorporeal shockwave therapy as part of the randomized controlled trial.

#### 2.6.2. Western-Blot

*(1) Protein Extraction and Quantification*. The total protein extracted from the serum of participants was homogenized in Laemmli sample buffer (2X) (Bio-Rad Laboratories) containing a protease inhibitor cocktail (Sigma-Aldrich). The lysates were centrifuged at 14,000 × *g* for 15 minutes at 4°C, and the supernatant was collected. Protein concentration was determined using the BCA Protein Assay Kit (Pierce Biotechnology) according to the manufacturer's instructions.

*(2) SDS-PAGE and Transfer*. Equal amounts of protein were separated by SDS-PAGE using 10% polyacrylamide gels, and then the transferring membrane marked the NC membrane in advance and equilibrated it in the precooled transfer membrane solution for a few minutes. The transfer film adopts the “sandwich type” to place the transfer film clip, sponge, transfer film filter paper, gel, NC membrane, transfer film filter paper, sponge, and transfer film clip in sequence and discharge air bubbles. After clamping the transfer film clip, press the positive and negative poles. Put the direction into the film transfer tank. The constant current is 220 mA, and the transfer time is adjusted according to the molecular weight of the protein.

*(3) Blocking and Antibody Incubation*. Transferring 5% skim milk to film, seal with NC film for 1-2 hours at room temperature. Rinse twice with 1 × TBST, tailor the NC membrane according to the desired protein molecular weight, and incubate the corresponding primary antibody at 4°C overnight. The next day, take out the NC membrane and rinse 4 times in 1 × TBST for 7 minutes each time. In this process, prepare a fluorescent secondary antibody or HRP-labeled secondary antibody and incubate the secondary antibody with the NC membrane for 2 h at room temperature (the fluorescent secondary antibody needs to be protected from light). Continue to rinse directly with 1 × TBST 4 times for 7 minutes each time.

*(4) Exposure Preparation of the Luminescent Substrate*. Mix the liquids A and B in the commercial ECL luminescence kit in equal volumes. After mixing, wrap it with thin foil to avoid light. Place the NC membrane on a clean plate, with the protein side facing up. Suck up the remaining TBST solution, and then slowly add the mixed ECL luminescent solution to make the luminescent solution evenly cover the surface of the film. Then, use the gel imaging system to take pictures and keep the data, and then use the corresponding software to analyze the optical density. The size of the optical density is positively correlated with the corresponding protein content.

*(5) Detection and Analysis*. Protein bands were visualized using the Enhanced Chemiluminescence (ECL) Detection System (Amersham Biosciences) and imaged using a ChemiDoc MP Imaging System (Bio-Rad). Band intensities were quantified using the Image Lab Software (Bio-Rad), and the intensity of Protein X was normalized to *β*-actin. Statistical analyses were performed using Student's *t*-test with significance set at *p* < 0.05.

### 2.7. Theme Flowchart

**Figure 2:**
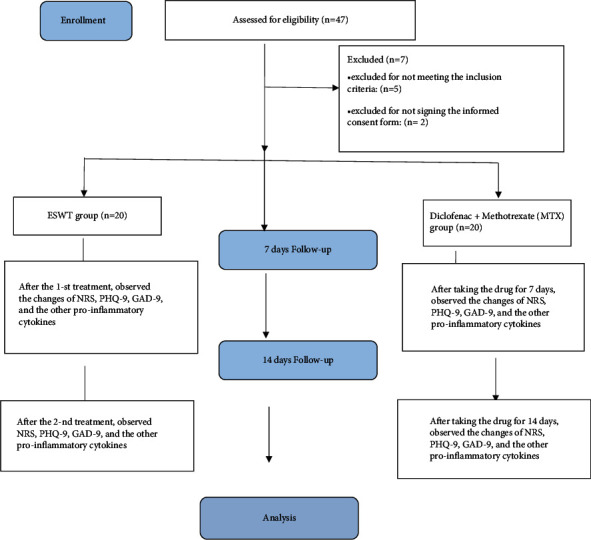
Theme flowchart.

The theme flowchart is presented in Figure 2.

### 2.8. Statistical Analysis

All data were collected at the Macau University of Science and Technology Hospital. All statistical analyses were performed by SPSS. Continuous data were expressed as mean ± standard deviation and analysed using independent sample *t*-tests, except for the analysis of internal cohorts, which were analysed using paired *t*-tests. Categorical data were expressed as the number of patients (*n*) and analysed by the chi-square test. Statistical differences were considered significant if the *p* value was <0.05.

## 3. Results

### 3.1. Clinical Characteristics of Patients

Prior to the initiation of the interventions, baseline equivalence between the two groups was meticulously assessed to ensure the validity of our comparative analyses. Basic case characteristics include age, sex, and body mass index (BMI), as well as clinical parameters such as disease duration. We enrolled 40 patients, of whom 11 were male and 29 were female, and assigned 20 patients each to the Diclofenac + MTX and ESWT groups. All subjects received the intended treatment and were analyzed for outcomes at each time point. The analysis strategy for this experiment used intention-to-treat to maximize adherence to randomization. The patient groups did not differ in baseline characteristics or outcome measures (Table 5). The mean age of patients was 41.38 ± 11.11 and 43.64 ± 12.27 years in the Diclofenac + MTX and ESWT groups, respectively. Most patients in both groups were female, 65% in the diclofenac + MTX group and 80% in the ESWT group. Both groups of the intent-to-treat population had moderate joint pain severity, with mean NRS scores of 4.80 in the ESWT group and 5.10 in the diclofenac/MTX group. Baseline characteristics of the participants were collected and analyzed prior to the start of the intervention. The mean age of the participants was 42.51 years, with a standard deviation of 12.91 years. Demographic characteristics, including age and gender distribution, were consistent with the general RA patient population, indicating a representative sample for this study.

Baseline disease characteristics such as duration of RA and numerical rheumatism score (NRS) were recorded. These parameters were compared with epidemiologic data from the broader RA patient community. The mean duration of RA in our study group was 8 years, which is consistent with the existing literature on RA progression, suggesting that our sample adequately represents the typical clinical course of the disease in the target population.

Regarding treatment history, all participants had previously received nonsteroidal anti-inflammatory drugs (NSAIDs) and disease-modifying antirheumatic drugs (DMARDs), with 60% having been treated with biologic agents, which is consistent with standard RA treatment in clinical practice.

### 3.2. Measurement of Pain Severity and Other Clinical Indicators

After the intervention, both groups in the intent-to-treat population experienced a decrease in pain levels during movement, as indicated by the NRS scale. Notably, the reduction in pain was greater in the ESWT group compared to that of the Diclofenac + MTX group across all time periods (*P* 0.05). In the ESWT group, the average NRS score dropped by 3.75 points after 14 days (from a baseline of 4.9 to 1.15 on day 14; *P* 0.05), whereas for the Diclofenac + MTX group, serving as the positive control, the average NRS score decreased by 2.2 points after 14 days (from 4.75 at baseline to 2.55 on day 14; *P* 0.05) (Table 6). Moreover, the majority of the patients were able to resume their normal daily activities or employment tasks.

**Figure 3:**
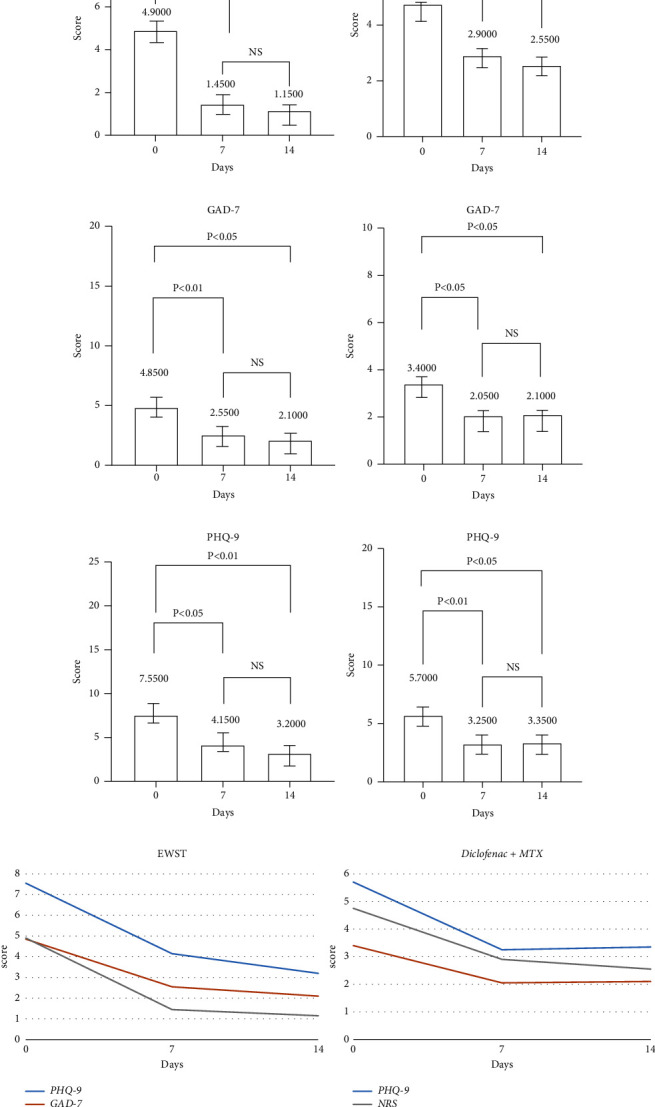
The result of pain severity and other clinical indicators (a–c), (d) line plots of changes in NRS, PHQ-9, and GAD-7.

Our clinical study revealed that both groups displayed decreased depression and anxiety assessment scores after undergoing 14 days of therapy, which was consistent with trends observed in pain levels in patients. This can be attributed to reduced pain intensity, leading to improved physical well-being and psychological relief that subsequently alleviates symptoms of depression and anxiety. It is worth noting that rheumatoid arthritis, an autoimmune disease characterized by chronic inflammation, can be managed through pharmacological treatments. These treatments reduce inflammation at a local and systemic level by decreasing the release of inflammatory mediators, which may also influence nervous system modulation, leading to eased depression and anxiety symptoms (Figure 3). A recent meta-analysis showed that anticytokine therapy can significantly improve depressive symptoms in inflammatory diseases. Importantly, no adverse events were reported throughout the 14-day clinical study.

### 3.3. Effect on the Expression of Serum Inflammatory Mediators and Protein Markers

**Figure 4:**
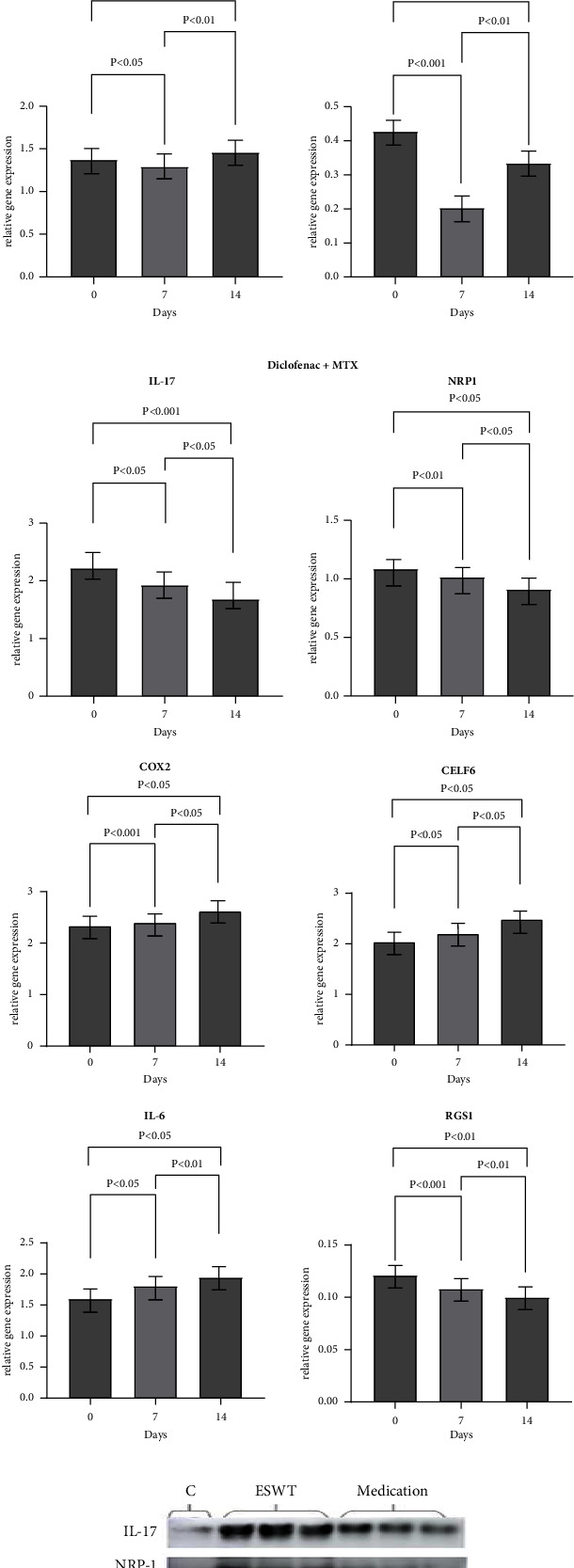
The result of real-time PCR (a, b), western blotting (c), and line plots (d) of changes in mRNA expression of the six target biomarkers during the course of the intervention.

The levels of biomarker proteins and inflammatory mediators in the venous blood of subjects were evaluated with western blot and real-time PCR. Both interventions demonstrated a similar trend in reducing inflammation levels among the patients (Figure 4). During the 14-day clinical trial, IL-17 mRNA expression consistently decreased in both patient groups, with a significant difference observed between the two treatment groups (ESWT: 2.63 ± 0.29, Diclofenac + MTX: 2.22 ± 0.07, *p*=0.028), indicating a reduction in inflammation. In the initial phase of the intervention (days 1–7), both groups demonstrated significant decreases in mRNA expressions of NRP-1, COX-2, and CELF-6. Particularly, the ESWT group showed a more pronounced effect in reducing COX-2 (ESWT: 1.90 ± 0.69, Diclofenac + MTX: 2.20 ± 0.09, *p*=0.015) and CELF-6 expression (ESWT: 1.29 ± 0.028, Diclofenac + MTX: 1.81 ± 0.002, *p*=0.006, respectively). However, there were no significant changes in the mRNA expressions of these three biomarkers during the second phase of intervention from days 7 to 14. Additionally, both IL-6 and RSG-1 mRNA expression showed a decrease followed by an increase in the ESWT group.

### 3.4. ESWT Promotes Tissue Repair More Rapidly in the Early Stage of Intervention

**Figure 5:**
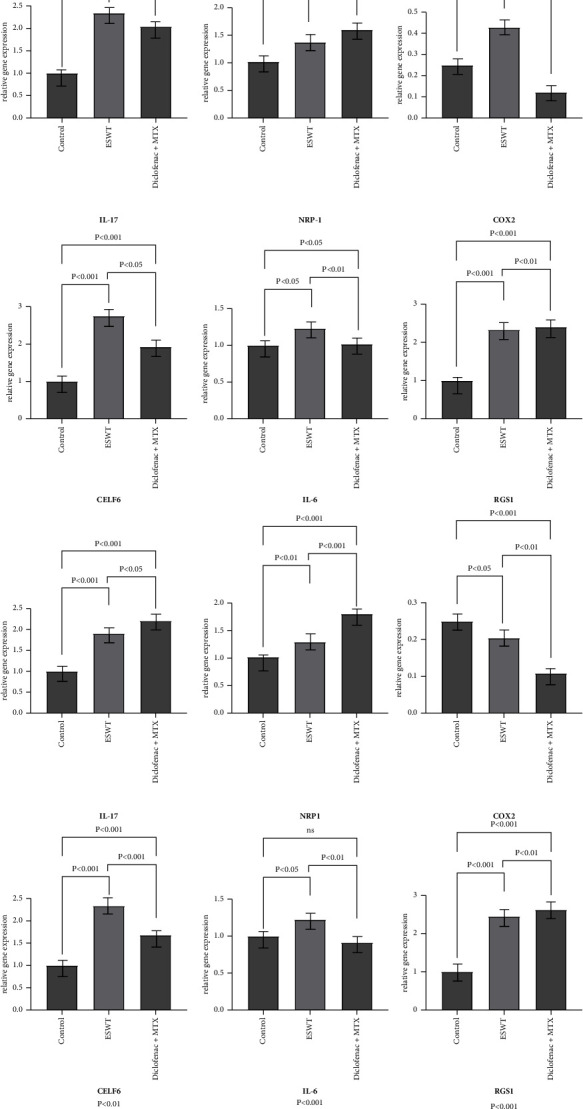
Expression of target biomarker mRNAs in venous blood of subject patients before the intervention was performed (a), at the end of the first phase of the intervention (b), and at the end of the second phase of the intervention (c). Differences were not considered statistically significant when p was greater than 0.05.

Figure 5 illustrates the mRNA expression of serum biomarkers across three time points during the clinical trial for both subject groups. Real-time PCR results showed elevated mRNA expression of inflammation-associated biomarkers in both patient groups, likely due to persistent inflammatory effects.

At the end of the first phase of the intervention (Figure 5(b)), no significant differences were observed in IL-17 and COX-2 mRNA expression between the two groups. However, compared to the Diclofenac + MTX group, patients that underwent ESWT intervention had significantly higher mRNA expression of NPR-1 (ESWT: 1.23 ± 0.06, Diclofenac + MTX: 1.02 ± 0.007, *p*=0.013) and RGS-1 (ESWT: 0.20 ± 0.01, Diclofenac + MTX: 0.11 ± 0.008, *p*=0.0001) in their blood samples. NPR-1 facilitates the migration of vascular endothelial cells, promotes angiogenesis, and aids in tissue repair by modulating the VEGF signalling pathway. Acting as a receptor protein, NPR-1 binds to VEGF and triggers the activation of the VEGF signalling pathway. This, in turn, stimulates the movement of endothelial cells towards the injured site, fosters the formation of new blood vessels, and contributes to the process of tissue repair. On the other hand, RGS-1 functions to mitigate the inflammatory response by negatively regulating G protein-coupled receptor signalling. By inhibiting such signalling, RGS-1 helps regulate the strength and duration of the inflammatory response. Therefore, it can be inferred that damaged tissues exhibit faster responses to ESWT compared to the positive control medications employed due to the accelerated actions of repair mechanisms and the modulation of inflammatory reactions.

Similarly, the mRNA expression levels of inflammation-associated IL-6 and CELF-6 differed between the two groups. The ESWT group had lower mRNA expression of IL-6 and CELF-6 compared to the Diclofenac + MTX group, with *P* values of 0.01 and 0.02, respectively. Overall, during the first phase of the trial, ESWT demonstrated a more significant reduction in inflammation and tissue repair compared to diclofenac combined with MTX therapy.

## 4. Discussion

Overall, the study aimed to compare ESWT with a routine drug regimen of Diclofenac and MTX over a 14-day treatment for RA patients. The results showed that ESWT was more effective in reducing pain than Diclofenac and MTX during the same period.

Inflammation represents a ubiquitous immune response of the injured body to external triggers, including pathogenic microorganisms, abnormal cells, chemical irritants, and various types of trauma (e.g., burns, radiation, and frostbite). Such inflammatory processes play a pivotal role in repairing, remodeling, and renewing different organs while also helping maintain the internal homeostasis of the tissue or system [15]. Diseases such as rheumatoid arthritis (RA) are closely linked with the onset and progression of uncontrolled inflammation. Therefore, an anti-inflammatory approach represents a highly promising strategy for preventing and treating RA and inflammation-related conditions such as atherosclerosis and diabetes mellitus [16].

To this end, small-molecule drugs such as glucocorticoids, nonsteroidal anti-inflammatory drugs (NSAIDs), and antioxidants have been frequently employed for the treatment of RA. Despite their widespread use, medications can cause various adverse effects, such as osteoporosis, aseptic joint necrosis, gastrointestinal bleeding, liver and kidney damage, and an increased risk of cardiovascular disease. The progress of medical technology has resulted in the use of monoclonal antibodies in clinical therapy and preclinical experimentation to modify inflammation-associated chemotactic molecules and cells in cases of RA. Despite their benefits, biologics have limitations due to their increased susceptibility to severe infections and malignancies.

In this trial, the experimental and control groups were comparable in baseline characteristics, with no significant differences observed in terms of age, gender, weight, other underlying conditions, NRS values, PHQ-9 scores, or GAD-7 scores. Clinical indicators highlighted substantial improvement in the shockwave group. ESWT group patients experienced a significant decrease in pain, reporting a mean NRS of 1.45 after 7 days of treatment and 1.15 after 14 days of treatment. Additionally, ESWT's regulatory effects on inflammatory factors and pain levels were evident in the change in blood biomarker protein levels, inflammatory cytokines, and psychiatric evaluation indicators of the patients, and such effects were sustained for 14 days, indicating a favourable and lasting therapeutic effect.

Although not as pronounced as the ESWT group, patients receiving Diclofenac + MTX also reported reduced pain, with a mean NRS score of 3.88 on day 7 after treatment, decreasing to 3.31 by day 14. While pain relief did not reach more than 50%, the overall well-being of patients significantly improved compared to pretreatment conditions. In contrast to the Diclofenac + MTX group, the shockwave group experienced a more significant reduction in the NRS score and yielded better patients' clinical assessment and overall response.

Previous research studies [17–19] have indicated that Extracorporeal Shock Wave Therapy (ESWT) has an anti-inflammatory effect, particularly on macrophages. In our research, we analyzed clinical questionnaires and blood samples from patients in the shockwave group. The results showed that more than half of the inflammatory factors, including CELF-6, IL-6, COX-2, and RGS-1, exhibited a down-regulation of the marker index after the ESWT treatment. Compared to anti-inflammatory drugs, ESWT demonstrated a more significant reduction in inflammation levels and facilitated tissue repair within the same intervention duration. These findings align with previous clinical trials conducted for bone and joint diseases [20]. A meta-analysis also indicated that ESWT was more effective than other nonsurgical treatments [21]. This may be attributed to ESWT's positive effect on local blood circulation, as confirmed by animal trials [22] and clinical trials [23], which subsequently benefits tissue nutrient supply [24].

Additionally, the study conducted by Calcagni et al. [25] provided evidence suggesting a slight proinflammatory impact of shock wave therapy. This finding has also been supported by studies utilizing burn wound models and ESW treatment [26–28]. Our findings further support the notion that shock waves may have a minimal proinflammatory effect.

The expression of NRP1 and nav2 protein in our data did not show any significant trend. Furthermore, our real-time PCR results showed elevated mRNA expression of inflammation-associated biomarkers in both patient groups. We were likely due to persistent inflammatory effects. But we should also consider the inflammatory response of ESWT to trigger. Particularly, polymorph nuclear neutrophil granulocytes are known to express a variety of angiogenesis-relevant factors and to have a proangiogenic impact. Even polymorph nuclear neutrophil granulocyte attachment to the activated endothelium before migration into the inflamed tissue stimulates the production of angiogenesis-relevant substances.

Not to mention, safety is paramount in therapeutic intervention, and numerous studies have evaluated the safety profile of ESWT and reported it as safe with minimal adverse effects. ESWT's degree of safety appears to correlate with indication and site of performance. For instance, a meta-analysis of randomized controlled trials for orthopaedic conditions confirms that ESWT found no adverse effects in any of the included studies [29]. Other single randomized controlled trials reported self-limiting adverse effects. For example, Gruenwald et al. [30] investigated the safety of ESWT for erectile dysfunction (ED). The researchers enrolled 156 patients and administered ESWT sessions over a span of nine weeks. They observed that most adverse events were mild and self-limiting, including penile hematoma in 3% of patients and mild pain during treatment in 2% of patients. These side effects disappeared shortly after the completion of treatment, suggesting a low risk associated with ESWT for ED.

While these studies provide evidence supporting the safety of ESWT, its administration should still be performed by qualified healthcare professionals following established protocols. Additionally, patient selection and appropriate dosing are crucial factors in minimizing adverse effects and optimizing outcomes.

## 5. Conclusion

Both treatments demonstrated substantial improvements in patients' joint function and reduced their pain and inflammation levels. Compared to the combination of diclofenac and MTX treatment, ESWT offered a more significant clinical analgesic effect and quicker, more profound reduction in inflammation during the early stages of treatment. This was achieved through the modulation of NRP-1, a receptor for trophic factors that promotes the movement of vascular endothelial cells and stimulates tissue repair through angiogenesis. Additionally, ESWT influenced RGS-1, which restricted the transmission of inflammatory signals and the activation of immune cells.

## 6. Limitations and Outlook

The study has limitations in measuring treatment outcomes. We aimed to evaluate levels of RA marker proteins and inflammatory markers in blood samples of RA patients before and after ESWT treatment to understand the therapeutic mechanism. However, our preliminary assessment only focused on levels of articular pain in patients and did not comprehensively evaluate joint function and ambulatory ability. Our study is limited by small sample size and short duration. Moreover, we did not assess long-term outcomes beyond the scope of this study.

For future studies exploring a broader application of ESWT, there are several notable directions to consider. ESWT's transition from experimental therapy to clinical application and the elucidation of its mechanism have necessitated prioritizing the assessment of its therapeutic efficacy. This can be achieved by incorporating clinical scales during the assessment of patients' motor function recovery. Additionally, gaining an understanding of how ESWT impacts the expression of wound healing-related markers at a molecular level can provide invaluable insights.

## Figures and Tables

**Table 1:** The 2010 American College of Rheumatology/European League Against Rheumatism classification criteria for rheumatoid arthritis.

Indicators	Score
*(A) Joint involvement*	
1 large joint	0
2−10 large joints	1
1−3 small joints (with or without involvement of large joints)	2
4−10 small joints (with or without involvement of large joints)	3
>10 joints (at least 1 small joint)	5

*(B) Serology (at least 1 test result is needed for classification)*	
Negative RF and negative ACPA	0
Low-positive RF or low-positive ACPA	2
High-positive RF or high-positive ACPA	3

*(C) Acute-phase reactants (at least 1 test result is needed for classification)*	
Normal CRP and normal ESR	0
Abnormal CRP or abnormal ESR	1

*(D) Duration of symptoms*	
<6 weeks	0
≥6 weeks	1

ACPA: anti-citrullinated peptide antibodies; CRP: C-reactive protein; ESR: erythrocyte sedimentation rate.

**Table 2:** Specific parameters of shockwave intervention.

Parameters	Data
Energy setting	0–20 levels
Wave frequency	0–8 Hz
Reachable depth	5–40 mm
Aperture angle	94°
Energy density	0.03–0.4 mJ/mm^2^
Pressure	11–82 MPa

**Table 3:** Severity grading of adverse reactions.

Severity	Features
Mild	Subjects/patients can perceive symptoms or signs, but it was easily tolerated
Moderate	Symptoms or signs that cause discomfort and interfere with daily activities
Severe	Subject/patient loses the ability to work or perform daily activities

**Table 4:** Primers used for RT-qPCR validation.

Genes	Sequence	Length	MW	Purification	nmol/OD
COX-2F	GCATTCTTTGCCCAGCACT	19	5714.67	OPC	6.02
CELF-6F	CCAGCCACAGAAAGAAACAGAAG	23	7077.54	OPC	4.10
IL-17F	CCATCTCATAGCAGGCACAAACT	23	6961.50	OPC	4.53
NRP-1F	GCAACGATAAATGTGGCGATACT	23	7096.52	OPC	4.30
RGS-4F	AGGAAGTCAAGAAATGGGCTGAA	23	7194.54	OPC	4.04
COX-2R	AAAGGCGCAGTTTACGCTGT	20	6156.89	OPC	5.16
CELF6R	GTCACTCCTCACCAGCCCTATTT	23	6870.44	OPC	4.96
IL-17R	GGATTTCGTGGGATTGTGATTCC	23	7116.45	OPC	4.58
NRP1R	TCGAAGTGAGGGTTGAAGTTGAT	23	7198.50	OPC	4.26

**Table 5:** Demographic and baseline clinical characteristics of patients in the intent-to-treat population.

Characteristics	Healthy people (*n* = 20)	ESWT (*n* = 20)	MTX + Diclofenac (*n* = 20)
Age (y)	40.90 ± 11.45	43.64 ± 12.27	41.38 ± 11.11
Women, *n* (%)	12 (60)	16 (80)	13 (65)
Weight (kg)	60.18 ± 9.32	60.93 + 8.04	61.77 ± 10.44
Height (cm)	168.34 + 8.18	161.75 + 6.80	166.01 + 8.97
BMI (kg/m^2^)	22.91 ± 3.26	23.28 ± 2.87	22.26 ± 2.08
PHQ-9	1.57 ± 0.33	6.79 + 4.36	9.00 + 5.11
GAD-7	0.64 ± 0.31	4.37 + 2.81	5.80 + 4.39
NRS	1.10 ± 0.55	4.75 + 1.50	4.9 + 1.23
Duration (years)	—	8.1 ± 2.21	7.9 ± 1.23

**Table 6:** Changes in subjects' anxiety and pain levels during intervention.

Days	NRS			PHQ-9			GAD-7		
	0	7	14	0	7	14	0	7	14
ESWT	4.9 ± 1.2	1.45 ± 0.9	1.15 ± 0.8	7.55 ± 4.9	4.15 ± 2.8	3.2 ± 2.3	4.85 ± 3.5	2.55 ± 2.4	2.1 ± 2.1
Diclofenac + MTX	4.75 ± 1.1	2.9 ± 0.9	2.55 ± 0.9	5.7 ± 3	3.25 ± 1.7	3.35 ± 1.9	3.4 ± 2.14	2.05 ± 1.4	2.1 ± 1.2

## Data Availability

Research data are not shared.

## References

[1] Guidelines A. C. O. R. S. O. R. A. (2002). Guidelines for the management of rheumatoid arthritis: 2002 update. *Arthritis & Rheumatism*.

[2] Rindfleisch A. J., Muller D. (2005). Diagnosis and management of rheumatoid arthritis. *American Family Physician*.

[3] Mehrpooya M., Majmasanaye M., Faramarzi F., Eshraghi A., Faress F. (2023). Investigation of the effect of oral selenium on the reduction of clinical symptoms and joint pain in patients with rheumatoid arthritis in the Iranian population. *The Journal of Clinical Pharmacology*.

[4] Walsmith J., Roubenoff R. (2002). Cachexia in rheumatoid arthritis. *International Journal of Cardiology*.

[5] Safiri S., Kolahi A. A., Hoy D. (2019). Global, regional and national burden of rheumatoid arthritis 1990–2017: a systematic analysis of the Global Burden of Disease study 2017. *Annals of the rheumatic diseases*.

[6] Khilfeh I., Guyette E., Watkins J., Danielson D., Gross D., Yeung K. (2019). Adherence, persistence, and expenditures for high-cost anti-inflammatory drugs in rheumatoid arthritis: an exploratory study. *Journal of Managed Care & Specialty Pharmacy*.

[7] Wong C. W. Y., Ng E. Y., Fung P. W., Mok K., Yung P. S., Chan K. (2017). Comparison of treatment effects on lateral epicondylitis between acupuncture and extracorporeal shockwave therapy. *Asia-Pacific Journal of Sports Medicine, Arthroscopy, Rehabilitation and Technology*.

[8] Liao C.-D., Xie G.-M., Tsauo J.-Y., Chen H.-C., Liou T.-H. (2018). Efficacy of extracorporeal shock wave therapy for knee tendinopathies and other soft tissue disorders: a meta-analysis of randomized controlled trials. *BMC Musculoskeletal Disorders*.

[9] Wang R., Li M., Ding Q. (2021). Neuron navigator 2 is a novel mediator of rheumatoid arthritis. *Cellular and Molecular Immunology*.

[10] Prieto D., González C., Weber L. (2021). Soluble neuropilin-1 in gingival crevicular fluid is associated with rheumatoid arthritis: an exploratory case-control study. *Journal of Oral Biology and Craniofacial Research*.

[11] Fan H., Liu G., Zhao C., Li X., Yang X. (2015). Differential expression of COX-2 in osteoarthritis and rheumatoid arthritis. *Genetics and Molecular Research*.

[12] Bi Y.-H., Wang J., Guo Z.-J., Jia K.-N. (2022). Characterization of ferroptosis-related molecular subtypes with immune infiltrations in neuropathic pain. *Journal of Pain Research*.

[13] Achudhan D., Liu S.-C., Lin Y.-Y. (2021). Antcin K inhibits TNF-*α*, IL-1*β* and IL-8 expression in synovial fibroblasts and ameliorates cartilage degradation: implications for the treatment of rheumatoid arthritis. *Frontiers in Immunology*.

[14] Aletaha D., Neogi T., Silman A. J. (2010). 2010 rheumatoid arthritis classification criteria: an American college of rheumatology/European league against rheumatism collaborative initiative. *Arthritis & Rheumatism*.

[15] Eshraghi A., Talasaz A. H., Salamzadeh J. (2016). Evaluating the effect of intracoronary N-acetylcysteine on platelet activation markers after primary percutaneous coronary intervention in patients with ST-elevation myocardial infarction. *American Journal of Therapeutics*.

[16] Atar A., Eshraghi A. A., Asgari S., GhA N., Badiee A. (2011). Antioxidant effect of ziziphus vulgaris, portulaca oleracea, berberis integerima and gundelia tournefortti on lipid peroxidation, Hb glycosylation and red blood cell hemolysis. *DOAJ (DOAJ: Directory of Open Access Journals)*.

[17] Mariotto S., de Prati A. C., Cavalieri E., Amelio E., Marlinghaus E., Suzuki H. (2009). Extracorporeal shock wave therapy in inflammatory diseases: molecular mechanism that triggers anti-inflammatory action. *Current Medicinal Chemistry*.

[18] Davis T. A., Stojadinovic A., Anam K. (2009). Extracorporeal shock wave therapy suppresses the early proinflammatory immune response to a severe cutaneous burn injury. *International Wound Journal*.

[19] Sukubo N. G., Tibalt E., Respizzi S., Locati M., d’Agostino M. C. (2015). Effect of shock waves on macrophages: a possible role in tissue regeneration and remodeling. *International Journal of Surgery*.

[20] Jhan S.-W., Wang C.-J., Wu K.-T. (2022). Comparison of extracorporeal shockwave therapy with non-steroid anti-inflammatory drugs and intra-articular hyaluronic acid injection for early osteoarthritis of the knees. *Biomedicines*.

[21] Mani-Babu S., Morrissey D., Waugh C., Screen H., Barton C. (2015). The effectiveness of extracorporeal shock wave therapy in lower limb tendinopathy: a systematic review. *The American Journal of Sports Medicine*.

[22] Kang N., Zhang J., Yu X., Ma Y. (2017). Radial extracorporeal shock wave therapy improves cerebral blood flow and neurological function in a rat model of cerebral ischemia. *American Journal of Tourism Research*.

[23] Schleusser S., Song J., Stang F. H., Mailaender P., Kraemer R., Kisch T. (2020). Blood flow in the scaphoid is improved by focused extracorporeal shock wave therapy. *Clinical Orthopaedics and Related Research*.

[24] Hashimoto S., Ichinose T., Ohsawa T., Koibuchi N., Chikuda H. (2019). Extracorporeal shockwave therapy accelerates the healing of a meniscal tear in the avascular region in a rat model. *The American Journal of Sports Medicine*.

[25] Calcagni M., Chen F., Högger D. C. (2011). Microvascular response to shock wave application in striated skin muscle. *Journal of Surgical Research*.

[26] Goertz O., Hauser J., Hirsch T. (2015). Short-term effects of extracorporeal shock waves on microcirculation. *Journal of Surgical Research*.

[27] Goertz O., Lauer H., Hirsch T. (2012). Extracorporeal shock waves improve angiogenesis after full thickness burn. *Burns*.

[28] Yancopouloš G. D., Davis S., Gale N. W., Rudge J. S., Wiegand S. J., Holash J. (2000). Vascular-specific growth factors and blood vessel formation. *Nature*.

[29] Schmitz C., Császár N. B., Milz S. (2015). Efficacy and safety of extracorporeal shock wave therapy for orthopedic conditions: a systematic review on studies listed in the PEDro database. *British Medical Bulletin*.

[30] Gruenwald I., Appel B., Kitrey N. D., Vardi Y. (2013). Shockwave treatment of erectile dysfunction. *Therapeutic Advances in Urology*.

